# Major-General Robert Maitland O’Reilly

**Published:** 1912-12

**Authors:** 


### OBITUARIES.

---

Major-General Robert Maitland O'Reilly died at his home in Washington, November 3, from uremia; aged 68. Dr. O'Reilly was Surgeon-General of the U. S. Army from 1902 to 1909.

